# Oral Lesion as Unusual First Manifestation of Multiple Myeloma: Case Reports and Review of the Literature

**DOI:** 10.1155/2014/529452

**Published:** 2014-11-25

**Authors:** A. Romano, M. S. Marescalco, Chiara Liardo, L. Villari, C. Vetro, C. Conticello, F. Di Raimondo, S. Ferlito

**Affiliations:** ^1^Department of Clinical and Molecular Biomedicine, Section of Haematology, University of Catania, Via Citelli 6, 95124 Catania, Italy; ^2^Ospedale Ferrarotto, Divisione di Ematologia, Via Citelli 6, 95124 Catania, Italy; ^3^Scuola Superiore di Catania, Via San Nullo 5 I, 95125 Catania, Italy; ^4^1st Section of Dentistry, Department of Surgery, University of Catania, Via Citelli 6, 95124 Catania, Italy; ^5^Division of Pathology, AOU “Policlinico-Vittorio Emanuele”, Via Plebiscito 628, 95100 Catania, Italy

## Abstract

Extramedullary plasmacytoma (EMP) and solitary bone plasmacytoma (SBP) represent a disease continuum through a multistage process of cell differentiation, survival, proliferation, and dissemination, strictly related to multiple myeloma (MM), the second most common hematological malignancy. Herein, we report two cases of recurrent oral plasmacytoma progressed to MM, in which the first clinical sign of a more widespread disease was limited to the mouth. Based on our experience, we recommend a strict workup for the differential diagnosis between EMP, SBP, and MM for patients with oral plasmacytoma, including radiological exam of the skeleton, magnetic resonance imaging (MRI) of the bone, and positive emission tomography (FDG-PET). MRI and possibly PET can all be used to more sensitively detect EM plasmacytoma sites.

## 1. Introduction

Multiple myeloma (MM) is a multifocal plasma cell proliferation in the bone marrow, in which neoplastic cells replace normal marrow cells, produce excess immunoglobulin, and infiltrate the bone. In a few cases, neoplastic plasma cells can be disposed in a localized mass within the bone, in a condition known as solitary bone plasmacytoma [1] (SBP) or outside thus involving soft tissues, in a condition defined as extramedullary plasmacytoma [2] (SEP). Clinical manifestations of SEP are generally not specific and may include swelling, pain, numbness, bleeding, mobile teeth, and xerostomia [3–5].

MM is defined by ≥10% of plasma cell infiltration in the bone marrow, ≥30 g/L of monoclonal protein and presence of hypercalcemia, renal insufficiency, anemia, and bone lytic lesions (identified by the acronym of CRAB symptoms) [6, 7]. Up to 90% MM patients complain of symptoms due to bone disease, defined as multiple destructive lytic lesions of the skeleton, including severe demineralization and osteoporosis through pathological fractures [8]. Involved areas include skeleton segments at high content of bone marrow such as skull, spine, sternum, vertebrae, pelvis, and hip. Jawbone is MM site in 30% cases [9, 10]. Oral lesions are seldom the first sign of disease [11–14].

Solitary bone plasmacytoma (SBP) is an area of lytic bone destruction in an otherwise asymptomatic patient, occurring generally in axial skeleton (70% in spine, seldom in the mouth [15, 16], including the temporomandibular joint [17, 18]), in absence of plasma cell infiltration of the bone marrow, without serum/urine M-protein. Patients complain of bone pain, and in critical cases, SBP may cause cord/root compression. High dosage radiotherapy (>5000 Gy) is often curative. However, lesions larger than 5 cm, immunoparesis, and persistence of paraprotein after radiotherapy are adverse prognostic factors for progression to MM [1].

Extramedullary plasmacytoma (SEP) occurs rarely (<1% of cases) and preferentially at level of head and neck [3], upper airways including nose-pharynx, tonsils, and paranasal sinus [4] and rarely at the mouth [5]. Like in SBP the M-protein is low or absent; plasma cell infiltration in the bone marrow is less than 5%, in absence of end-organ damage and diffuse osteolytic lesions [2].

Here we report two cases of oral plasmacytoma, in which the first clinical sign of a more widespread disease was limited to the mouth.

## 2. Case A 

A 72-year-old Caucasian male presented to dental division complaining of pain and difficulty in chewing due to an overgrowth of soft tissue located in the alveolar ridge of the left mandible. He reported the extraction of the residual roots of 3.7 which occurred two weeks before. After two days, the alveolar mucosa grew up over the postextraction site. No pain or alterations of gingival mucosa were reported before the extraction of residual roots.

Extraoral examination revealed normal facial morphology, in absence of lymph node swelling. The orthopantomography did not show any sign of bone involvement. At intraoral examination, a soft mass was present (1.5 cm) with ill-defined contours being pink in colour and slightly ulcerated on top for the action of antagonist dental elements.

**Figure 1:**
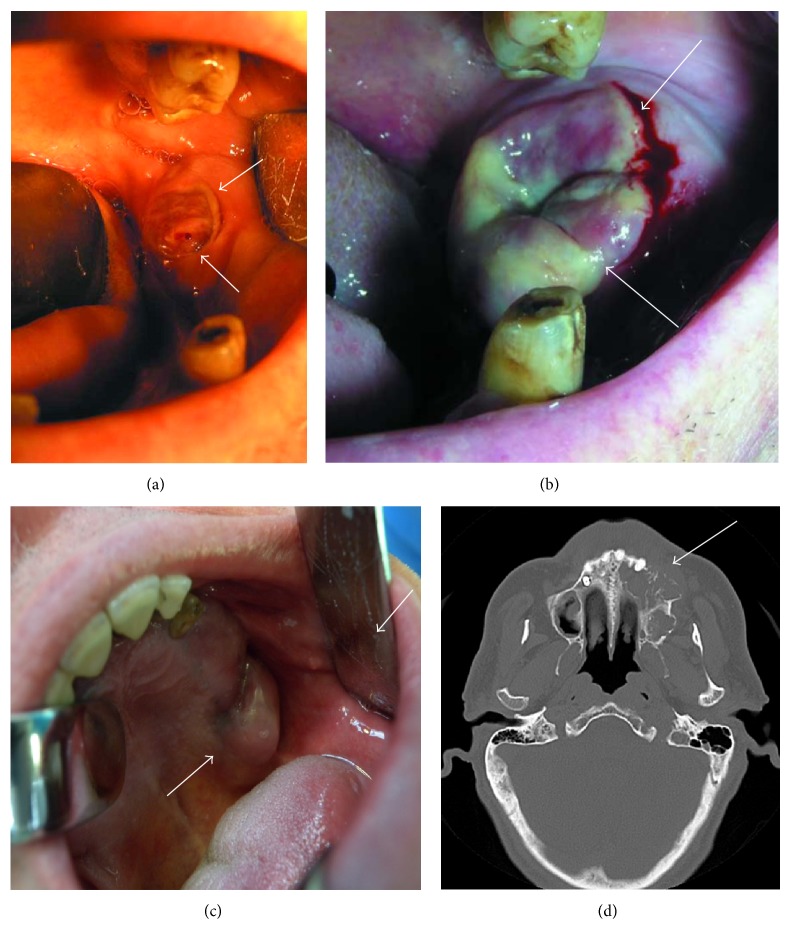
Extramedullary plasmacytoma, macroscopic features. (a) The soft mass neoplasm appears pink in colour and has floating consistency with a diameter of about 1.5 cm, occupying the area of dental elements 37 and 38. It has ill-defined contours and is slightly ulcerated on the top for the effects of antagonist dental elements, causing algia and difficulty in chewing for the patient. No sign of bone involvement at the orthopantomography (Case A). (b) The patient presented a relapse of the neoplasm seven days after the biopsy, with the enlargement of the lesion to the entire edentulous left side of the jaw, until the first premolar. The mucosa covering the lesion was of irregular colour and the traumatism due to the antagonist dental elements was accentuated (Case A). (c) The mass presented as a swelling in the left posterior region of the maxilla, with maximum dimensions 25 × 45 mm, involving also palate and gingiva. The mass consistency was hard elastic and normal in colour and covered with intact oral mucosa (Case B). (d) The maxillofacial CT scan showed an important osteolytic involvement of the upper alveolar process and the partial obliteration of the basal portion of the left maxillary sinus. The lesion extended also to the homolateral nasal cavity. Concomitant enlargement of lateral cervical and submandibular lymph nodes, with the biggest having 13 mm diameter (Case B).

**Figure 2:**
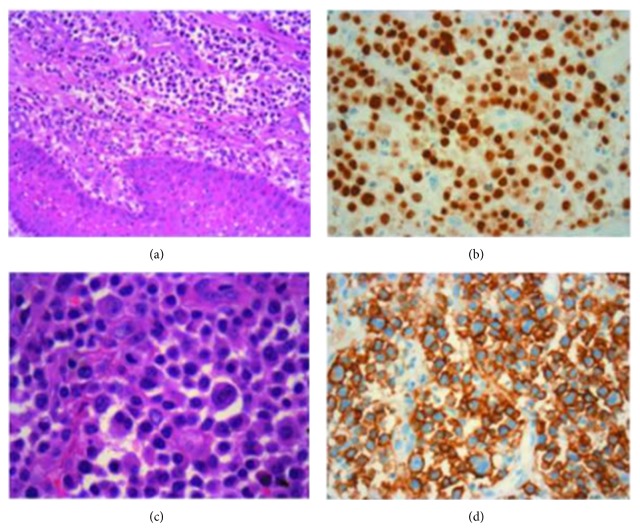
Extramedullary plasmacytoma, microscopic features. (a) The submucosa was heavily infiltrated by atypical plasma cells (hematoxylin eosine stain × 20, Case A). (b) MUM-1 + atypical plasma cells (nuclear staining pattern) (×40). (c) Some of the neoplastic plasma cells (pleomorphic or showing atypical mitosis stain × 63, Case B). (d) CD138 + atypical plasma cells (membranous staining pattern, ×40, Case B).

After the patient provided written informed consent, the soft tissue was biopsied. The histological examination revealed a granulation tissue dissociated by lymphoplasmacytic infiltrate. Seven days after the biopsy the soft mass recurred. Three days later, an excisional biopsy was performed (Figure 1). Histological analysis showed the presence of a poorly differentiated plasma cell neoplasm. Microscopically the tissue appeared heavily infiltrated by neoplastic proliferation with widespread growth, consisting of elements of plasmablastic/plasmacytic morphology and some of them frankly pleomorphic and anaplastic (Figure 2), compatible with the diagnosis of extramedullary plasmablastic/anaplastic plasmacytoma CD138+, CD38+, EMA+, vimentin+, CD79a weak, CD20−, and CD3−. The patient was therefore sent to the department of haematology for inquiries regarding the stage of disease and for subsequent treatment.

According to medical history, the patient was diabetic, asthmatic smoker. A comprehensive metabolic panel and complete blood count (CBC) revealed a total protein of 8.2 g/dL, due to a concomitant low amount of iron, without immunoparesis, in absence of a detectable serum or urine paraprotein by immunofixation. The bone marrow biopsy was negative for clonal plasma cells.

The patient underwent bone imaging: the skeletal survey was negative for osteolytic lesions; the fluorodeoxyglucose-positron emission tomography (FDG-PET) scan was weakly positive at level of mandible and the second cervical vertebra (SUVmax = 2.5), otherwise normal. This finding was confirmed at magnetic resonance imaging (MRI), otherwise normal; thus we considered these findings without an oncologic meaning.

In absence of CRAB criteria, SEP was diagnosed and the patient received radiotherapy at level of mandible and vertebra. The treatment was complicated by swelling, weight loss, and worsening asthenia.

Three months later, FDG-PET scan was repeated showing persistence of positivity, but in absence of symptoms.

Further three months later, clinical conditions worsened; magnetic resonance imaging (MRI) showed nodular lesions with intense enhancement in vertebrae, in the pelvic bone, and in the sternum. FDG-PET scan showed diffusely positive signal in the left jaw, sternum, and ankle, confirming the systemic nature of the disease. The bone marrow biopsy showed an infiltration of 30% monoclonal plasma cells.

In absence of circulating monoclonal component in serum and urine, with evidence of immunoparesis, nonsecretory MM was diagnosed, stage II in accord with International Staging System [24]. Laboratory findings are reported in Table 1.

Two months later, at eight months from the first occurrence of oral plasmacytoma, the patient started systemic chemotherapy with bortezomib 1.3 g/mq at days 1, 4, 8, and 11, thalidomide 100 mg for 21 days, and dexamethasone 40 mg weekly without benefit. The patient died of pneumonia two months later.

## 3. Case B

A 63-year-old Caucasian man, affected by monoclonal gammopathy of undetermined significance (MGUS) since five years, presented with an evolving swelling in the maxilla, at level of left posterior region with involvement of palate and gingiva. At the intraoral examination, the mass consistency was hard elastic and normal in color and covered with intact oral mucosa (Figure 1(c)).

The maxillofacial computerized tomography (CT) scan confirmed the presence of a solid neoplasia, with maximum dimensions 25 × 45 mm, causing a structural rearrangement of the bone. An osteolytic involvement of the upper alveolar process and the maxillary sinus was evident, which appeared partially obliterated in its basal portion. The neoplasm was extended to the homolateral nasal cavity with hyperdense swelling at the nose-maxillary region adjoining. Later enlarged cervical and submandibular lymph nodes were evident; the largest had 13 mm diameter.

The patient reported that the presence of soft tissue in the same site occurred around six months before and was already biopsied, compatible with diagnosis of mucosal plasmacytoma. The histological revision in our division confirmed the low-grade nature of that lesion (weak plasma cell infiltration, presence of small necrosis foci, low proliferation index, 3-4%, immunophenotype: CD138+, CD38+, EMA+, vimentin+, CD79a weak, CD20−, CD3−, CD45RO−, SMA−, myosin−, and S-100−, Figure 2). Hematological restaging confirmed persistency of MGUS, with immunofixation positive for IgA, stable M-protein (2200 mg/dL), no end-organ damage signs, including bone evaluation with X-ray scan and MRI, and 6% plasma cell infiltration in the bone marrow. Since the absence of organ damage (see biochemistry Table 1) and CRAB symptoms, the patient remained in follow-up without starting therapy.

Because of the recurrence of oral lesion two months later, new incisional biopsy was given. Histological examination of the 1.5 cm soft tissue fragment showed a massive component of well-differentiated plasma cells, secreting monotypic immunoglobulins with lambda light chain restriction.

Diagnosis of MGUS progression to extramedullary myeloma stage II was stated. The patient started systemic chemotherapy with lenalidomide 25 mg and dexamethasone 40 mg weekly, achieving partial remission. He is currently under maintenance therapy with 5 mg lenalidomide daily, in good clinical conditions.

Whereas thalidomide and lenalidomide might be of help in high-risk and EM-myeloma, most often, proteasome inhibitors, such as bortezomib, have more often been described to induce response and seem the better option to use.

## 4. Discussion

Herein, we report two cases of MM patients with unusual extramedullary sites (mouth) in which the systemic disease was anticipated by localized mouse disease. Both patients received first localized treatment (only radiotherapy) and strict follow-up, thus to shift to systemic chemotherapy later.

Maxillofacial lesions are very rare as first myeloma manifestation (<0.5%) and are more frequent in advanced disease with extensive skeletal involvement, including the skull [14]. A Japanese series reported oral manifestations of MM in almost 50% of initially diagnosed mouth plasmocytoma [18].

At X-ray scans, osteolytic lesions in maxilla appear well defined, without any sign of bone reaction [10], more frequent in the jaw than in the maxilla [14, 25].

**Figure 3:**
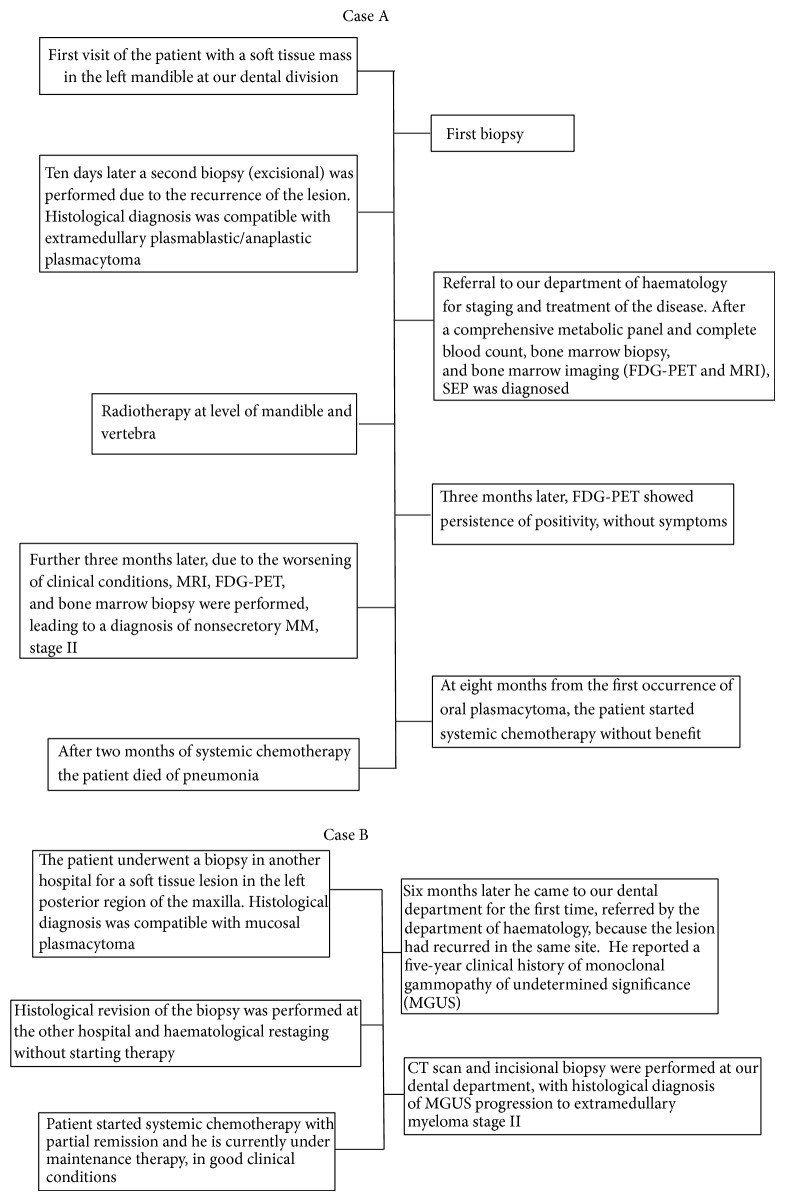


As shown in Case A (Figure 3), evolution of SEP is unpredictable. Once it is diagnosed and treated, the patient may have no symptoms for months or even years [26]. However, in unfavourable cases, the tumour may spread, developing regional localizations in 35–50% of cases, or evolve into multiple myeloma [26].

In patient B, SBP was the first clinical sign of MGUS progression through MM. Clinical investigations conclude that SBP is more likely to transform in MM than SEP, with a period of progression variable from months to a few years [9]. In the Mayo Clinic's series [12] including 33 patients affected by SBP, 43% of them converted to MM after a median of 20.7 months.

As shown in this report, localized PCN can be the first sign of systemic disease, including nonsecretory MM.

Thus, whoever is involved in dental care should search for MM signs in each patient with mouth plasmacytomas, including bone scan and if clinically indicated MRI and/or FDG-PET. The integrated use of MRI and FDG-PET can help clinicians to exclude other extramedullary localizations [27, 28]. Patients affected by extramedullary MM, both primary and progressing from SBP/SEP, have still a poor outcome despite the success of novel agents, due to acquired molecular lesion, including TP53 mutations [29, 30].

Preliminary observations in extramedullary MM have shown no efficacy of thalidomide [31] and controversial results for bortezomib [32, 33], more often associated with relapse with extramedullary spreading. Encouraging reports arise from lenalidomide based regimens, as our Case B confirms.

Whereas IMIDs (thalidomide, lenalidomide) might be of help in high-risk and EM-myeloma, most often, proteasome inhibitors, such as bortezomib, have more often been described to induce response and seem better to use.

The incidence of EMPs is 7% to 18% at MM diagnosis and up to 20% at relapse. The current notion that EMPs are more frequent after treatment with novel agents remains to be proven, especially considering that different patterns of disease recurrence can emerge as patients live longer in the era of novel drugs. Possible mechanisms of extramedullary spread include decreased adhesion molecule expression and downregulation of chemokine receptors. High-dose therapy with autologous stem-cell transplantation (ASCT) can overcome the negative prognostic impact of extramedullary disease in younger selected patients. EMPs do not typically respond to thalidomide alone, but, in contrast, responses to bortezomib have been reported. A potential first-line treatment option seems to be a bortezomib-containing regimen followed by ASCT, whenever possible [34].  Table 2 summarizes recent reports about outcome of EM-myeloma treated with bortezomib, where overall responses can achieve up to 50% [19–23].

## 5. Conclusions

SBP and SEP can evolve to MM. MM signs should be evaluated in each patient with mouth plasmacytomas, including bone scan and if clinically indicated MRI and/or FDG-PET. When SBP and SEP progress to MM, novel agents including lenalidomide and bortezomib are safe and efficacious in disease management.

## Figures and Tables

**Table 1:** Clinical variables at diagnosis of patients A and B.

	Case A	Case B	Normal range
Hemoglobin, g/L	10.5	10.6	12.1–15.1
Serum lactate dehydrogenase, U/L	377	256	256–450
Serum beta-2 microglobulin, mg/L	2.8	2.2	1.2–2.4
Serum albumin (g/dL)	4.0	4.1	3.5–5.0
Serum AST (U/L)	14	15	5–42
Serum ALT (U/L)	42	31	5–42
Serum calcium (mmol/L)	2.22	2.40	2.15–2.55
Serum creatinine (micromol/L)	108	89	60–125
Blood urea nitrogen (mmol/L)	5.2	4.8	2.5–8.0
ISS stage	I	I	—
Durie and Salmon Stage	1	1	—

**Table 2:** 

Number of patients	Regimen description	Response rate	Notes	Reference
275 54 w EMP	Bortezomib based + ASCT	Not reported	Retrospective series EMP is an independent factor for PFS and OS only in transplant-ineligible patients	Lee et al. [19]

1	(1) VTD + ASCT (2) Bortezomib + tanespimycin	Not reported	Case report	Kumar et al. [20]

97 (plasmacytoma evolved to MM)	Radiotherapy versus novel agents/chemotherapy	91.8%	Retrospective series Immunoparesis was the only negative predictor of progression to MM	Katodritou et al. [21]

36 (EMP with invasion of the spinal cord)	Bortezomib based	13/36 (including complete remission)		Zhang and Zhong [22]

50 (intracranial MM)	Bortezomib based (*n* = 29)	18/29 (CR + VGPR)	Retrospective series	Gozzetti et al. [23]
